# Exploring the Impact of Solid-State Fermentation on Fava Bean Flour: A Comparative Study of *Aspergillus oryzae* and *Rhizopus oligosporus*

**DOI:** 10.3390/foods13182922

**Published:** 2024-09-15

**Authors:** Ophélie Gautheron, Laura Nyhan, Maria Garcia Torreiro, Ali Zein Alabiden Tlais, Claudia Cappello, Marco Gobbetti, Andreas Klaus Hammer, Emanuele Zannini, Elke K. Arendt, Aylin W. Sahin

**Affiliations:** 1 School of Food and Nutritional Sciences, University College Cork, T12 YN60 Cork, Ireland; o.gautheron@umail.ucc.ie (O.G.); lnyhan@ucc.ie (L.N.); e.zannini@ucc.ie (E.Z.); aylin.sahin@ucc.ie (A.W.S.); 2 Mogu srl, Via S. Francesco, 62, 21020 Inarzo, VA, Italy; mgt@mogu.bio; 3 Faculty of Agricultural, Environmental and Food Sciences, Free University of Bozen-Bolzano, Piazza Università, 1, 39100 Bolzano, BZ, Italy; alizeinalabiden.tlais@unibz.it (A.Z.A.T.); claudia.cappello@unibz.it (C.C.); marco.gobbetti@unibz.it (M.G.); 4 Fraunhofer Institute for Molecular Biology and Applied Ecology, Ohlebergsweg 12, 35392 Giessen, Germany; andreas.hammer@ime.fraunhofer.de; 5 Dipartimento di Biologia Ambientale, Sapienza Università di Roma, 00185 Rome, RM, Italy; 6 APC Microbiome Ireland, University College Cork, T12 YT20 Cork, Ireland

**Keywords:** aroma, metabolites, nutrition, techno-functionalities, antinutritional factors, fava bean flour, solid-state fermentation

## Abstract

Fava bean (*Vicia faba* L.) is a protein-rich pulse with high nutritional value, but its functional and sensory characteristics limit its application in foods. Solid-state fermentation (SSF) can modify the composition of plant proteins, modulate its functionality, and enhance the sensory aspects. In this study, fava bean flour (FB) was fermented with *Aspergillus oryzae* and *Rhizopus oligosporus* to produce FBA and FBR, respectively, ingredients with distinct nutritional, functional, and aroma characteristics. The protein content increased by 20% in FBA and 8% in FBR, while fat levels rose more significantly in FBR (+40%). The overall content of fermentable oligo-, di-, mono-saccharides, and polyols (FODMAPs) decreased by 47% (FBA) and 57% (FBR), although polyol production by *A. oryzae* was observed. SSF improved the nutritional profile of FBA and FBR, with a notable increase in the concentration of essential amino acids observed, and a reduction in most antinutrients, with the exception of trypsin inhibitors. SSF resulted in the formation of aggregates, which increased the particle size and reduced protein solubility. Emulsions prepared with the fermented ingredients separated faster, and the foaming capacity of both FBA and FBR was decreased, but an increase in water-holding capacity was observed. SSF resulted in the production of predominantly savoury-associated aroma compounds, with compounds characteristic of metallic and mouldy aromas reduced. These results indicate the potential of SSF to transform FB with enhanced nutritional value and improved sensory and functional properties.

## 1. Introduction

Pulses are widely consumed as staple foods and serve as a crucial source of dietary protein for a large proportion of the world’s population, especially in regions where the consumption of animal protein is restricted due to limited availability or avoided due to religious or cultural practices [1].

Fava bean (*Vicia faba* L.) is one of the most widely grown pulses, following soybean and pea in terms of area and production [2]. It is recognised as a source of protein, accounting for 27–40%, with a low fat content (1–3%), composed mainly of oleic acid (monounsaturated fatty acid) and linoleic acid (polyunsaturated fatty acid) [3,4]. Fava bean also contains around 13% dietary fibre and 40% starch, as well as essential vitamins and minerals (primarily zinc, potassium, and iron) [3,4]. Including pulses in the diet may have potential health benefits, such as reducing the risk of cardiovascular disease by preventing hypertension and hypercholesterolemia [5]. However, their use in food is still limited, as they contain several bioactive compounds traditionally classified as antinutrients. These antinutritional factors include phytic acid, saponins, tannins, trypsin inhibitors, and flatulence-causing oligosaccharides [6]. They can have negative effects by reducing the digestibility of proteins and carbohydrates, and interfering with minerals’ bioavailability [7]. However, processing methods such as dehulling, soaking, cooking, fermentation, and germination can enhance the nutritive value of food legumes by reducing these effects [8].

Solid-state fermentation (SSF) has been cited in numerous studies for its ability to degrade antinutritional compounds, and in enhancing the sensory, compositional, and functional properties of legumes [9,10,11,12,13]. During SSF, the fermenting microorganism grows on a damp, solid substrate with a significantly low water content, allowing close contact between the microorganism and the gaseous oxygen from the air [14]. During SSF, microorganisms produce enzymes such as amylases, proteases, and lipases, resulting in the breakdown of macronutrients into more digestible compounds, which can enhance the aroma, flavour, and texture [13].

Filamentous fungi such as *Aspergillus oryzae* and *Rhizopus oligosporus* are used in industry to manufacture antibiotics, organic acids, and commercial enzymes [15]. *A. oryzae* has been used for centuries in Asia to make traditional fermented soy products, while *R. oligosporus* was traditionally used in Indonesia to produce tempeh by fermenting soybeans [16,17]. Both genera are considered as generally recognised as safe (GRAS) and have been applied in several studies on SSF of pulses [18,19]. Chawla et al. (2017) used SSF on black-eyed pea with *A. oryzae* and observed positive changes in functional properties such as water- and oil-holding capacities, emulsion and foaming properties, and the enhanced bioavailability and digestibility of iron and zinc [18]. Another study conducted by Toor et al. (2022), investigating the fermentation of different legumes (chickpea, pigeon pea and soybean) by *R. oligosporus*, revealed an increase in protein, ash, and amino acid contents. In addition, the SSF process changed the colour and some functional properties [19].

The aim of this study was to examine the effects of SSF with *A. oryzae* and *R. oligosporus* on the nutritional composition of fava bean flour. Furthermore, the research explored changes in the techno-functional properties and aroma characteristics of the ingredients.

## 2. Materials and Methods

### 2.1. Raw Materials and Starter Cultures

Fava bean flour (FB) (Müller’s Mühle, Gelsenkirchen, Germany) was fermented (Mogu, Inarzo, Italy) with *Aspergillus oryzae* (from a commercial koji starter from Starter Cultures, Amsterdam, The Netherlands) or *Rhizopus oligosporus* (from a commercial tempeh starter from Top Cultures, Zoersel, Belgium). The starters were prepared according to Chutrtong and Bussabun (2014) [20], with modifications. Briefly, clear sporulation on MYA medium was lyophilised (alpha 1-2 LDplus lyophiliser, 230 V, CHRIST, Germany) and mixed with sterilised rice flour (*Oryza sativa*, Riseria d’Italia S.r.l.), in a 9:1 weight ratio, using a laboratory mill. The starter was then stored in Mogu’s (Inarzo, Italy) growing room, which is equipped with a temperature-controlled AC system, for up to 60 days at 25 °C.

Reagents were purchased from Sigma-Aldrich (St. Louis, MO, USA), unless otherwise stated.

### 2.2. Solid-State Fermentation

The fava beans were fermented according to Gautheron et al. (2024) [21]. Briefly, the fava bean substrate was washed to remove any unwanted contaminants or residues that may have negatively impacted fermentation [22], and rehydrated by immersion in tap water (in a 1:2 ratio of substrate to tap water by volume) for 15 min. After draining, the substrate was autoclaved (121 °C for 15 min) and subsequently cooled to 30 °C.

The *R. oligosporus* tempeh-like fermentation was performed according to Erkan et al. (2020) with slight changes [23]. For pH adjustment to 4.5–5.5, 25 mL of wine vinegar was added to 500 g of the dry substrate during the cooling phase. For inoculation, 1.5 g of the tempeh starter was added to 500 g of the dry substrate and mixed thoroughly to ensure the homogenous distribution of spores. The substrate was tightly packed onto stainless steel trays (3.8 L volume) with a polycarbonate lid. Inoculation was performed manually under a laminar flow hood, keeping clean conditions but not completely sterile. The substrate and inoculum were mixed manually.

The *A. oryzae* koji-like fermentation was performed according to the method described by Kim et al. (2012) [24]. For this, 1.5 g of the koji starter was added to 500 g of the substrate, mixed thoroughly, and compacted onto stainless steel trays (3.8 L volume) with a polycarbonate lid. Inoculation was performed manually under a laminar flow hood, keeping clean conditions but not completely sterile. The substrate and inoculum were mixed manually.

The inoculated substrates were incubated on heating mats (24 × 52 cm, 220 V, Lerway) at 28 °C (tempeh) or 30 °C (koji), controlled by a thermostat (ITC-308, 220 V, Inkbird, Shenzen, China) which was placed into the middle of the substrate bed. For *A. oryzae*, the substrate was mixed 24 h after inoculation to ensure complete colonisation, dissipate heat, and promote aeration. After 48 h (for *R. oligosporus*) and 72 h (for *A. oryzae*), the substrate’s surface was covered with mycelium, forming a compact cake. The fermented substrate was freeze-dried, then ground into flour (particle size ≤ 100–200 μm).

### 2.3. Compositional Analysis

Compositional analysis was performed as described by Gautheron et al. (2024) [21].

#### Carbohydrates

Starch (resistant, digestible, and total) was determined using the Megazyme kit K-RAPRS (Megazyme, Bray, Ireland). Dietary fibre was determined using the K-RINTDF method (Megazyme, Bray, Ireland). Sugars (glucose, fructose, sucrose, maltose, and galactose), and FODMAPs (polyols: arabitol, sorbitol, and mannitol; oligosaccharides: raffinose/stachyose, verbascose, kestose, and nystose)were extracted as described by Ispiryan et al. (2019), and separated and quantified via high-performance anion-exchange chromatography coupled with pulsed amperometric detection (HPAEC-PAD) on a Dionex^TM^ ICS-5000+ system (Thermo Scientific, Sunnyvale, CA, USA) [25]. All carbohydrate measurements were performed using authentic reference standards.

### 2.4. Protein Characteristics

#### 2.4.1. Total Amino Acids and Free Amino Acids

Total amino acids were assessed following hydrolysis under acidic, oxidative-acidic, or alkaline conditions using a Sykam S433 amino acid analyser (Fürstenfeldbruck, Germany). External calibration was applied, following the method outlined by Ahlborn et al. (2019) [26]. The true protein content was calculated by summing up the amino acid residues, factoring in the added water amount for peptide bond hydrolysis.

Free amino acid concentrations were determined by MS-Omics Aps (Vedbæk, Denmark). The ingredients underwent derivatisation with methyl chloroformate, following a slightly modified version of the protocol outlined by Smart et al. (2010) [27], as reported by Gautheron et al. (2024) [21]. 

#### 2.4.2. Sulfhydryl (SH) Groups

Exposed, free, and total SH groups were quantified using Ellman’s method, as described by Gautheron et al. (2024) [21]. 

#### 2.4.3. Protein Solubility

Protein solubility was measured at the native pH and pH 7 using the Kjeldahl method (AACC Method 46-12.01 [28]), as described by Jaeger et al. (2023) [29]. 

#### 2.4.4. SDS-PAGE

The protein profile of the ingredients was determined by SDS-PAGE, as described by Gautheron et al. (2024) [21]. 

### 2.5. Techno-Functional Properties of the Ingredients

#### 2.5.1. pH and Total Titratable Acidity (TTA)

The pH and TTA of the ingredients were assessed following the method outlined by Jaeger et al. (2023) [29]. 

#### 2.5.2. Water- and Oil-Holding Capacity

Water-holding capacity (WHC) and oil-holding capacity (OHC) were measured in accordance with the method of Boye et al. (2010), with slight modifications [1], as described by Gautheron et al. (2024) [21].

#### 2.5.3. Foaming Properties

For this, 2% (*w*/*w*) dispersions of the ingredients were prepared in distilled water, and foaming capacity and foam stability were assessed according to Gautheron et al. (2024) [21].

#### 2.5.4. Minimum Gelling Concentration

The minimum gelling concentration was determined following the method of Vogelsang et al. (2020) [30]. 

#### 2.5.5. Emulsion Characteristics

Emulsion stability was evaluated using the method described by Jaeger et al. (2023) [29].

#### 2.5.6. Particle Size

The particle size distribution of the protein ingredients was measured by laser diffraction using the Mastersizer 3000 (Malvern Instruments Ltd., Worcestershire, UK) equipped with the AERO-S attachment. The refractive index of the particles was set to 1.45, and the absorption index was set to 0.001.

#### 2.5.7. Colour

The ingredients’ colour was measured using a ChromaMeter CR-400 (Konica Minolta, Osaka, Japan), based on the CIE L*a*b* colour space system, as described by Gautheron et al. (2024) [21]. The differential colour index value was calculated using the following equation

(1) $$∆E=\sqrt{({∆L}^{*}{)}^{2}+({∆a}^{*}{)}^{2}+({∆b}^{*}{)}^{2}}$$

where $∆L^{*}=L_{FB}^{*}-L_{FBA/FBR}^{*}$, $∆a^{*}=a_{FB}^{*}-a_{FBA/FBR}^{*}$, and $∆b^{*}=b_{FB}^{*}-b_{FBA/FBR}^{*}$

### 2.6. Antinutrients

Antinutritional factors of the ingredients were determined as follows. All results are expressed as dry matter and were analysed in duplicate.

#### 2.6.1. Phytic Acid

Phytic acid concentrations were measured using a phytic acid (phytate)/total phosphorus kit (Megazyme International, Ireland), which quantified the phosphorus released by the enzymatic action of phytases. Briefly, 1 g of each ingredient was mixed with 20 mL of 0.66 M HCl and incubated overnight at 25–28 °C. The ingredients were then centrifuged for 10 min at 18,516 rcf, and the pH of the supernatant was adjusted to 7 using 0.75 M NaOH. The extracts were processed to determine the concentrations of free and total phosphorus using a phosphorus calibration curve, as per the manufacturer’s instructions. The phosphorus content was used to estimate the phytic acid concentrations via a mathematical formula provided in the instruction manual.

#### 2.6.2. Total Saponin

The total saponin content was quantified using a modified version of the method by Lai et al. (2013), as detailed by Krause et al. (2023) [31,32]. Briefly, 0.5 g of each ingredient was defatted using 10 mL of petroleum ether by continuous shaking for 4 h. After evaporation of the solvent, 20 mg of the residues were extracted by mixing with 5 mL of 80% (*v*/*v*) methanol for 4 h. The mixture was centrifuged at 7916 rcf for 10 min at 4 °C, and the supernatants were stored in the dark at 4 °C until use. To prepare the assay, 0.1 mL of the ingredient’s extract, 0.4 mL of 80% (*v*/*v*) methanol, 0.5 mL of a freshly prepared 8% ethanolic vanillin solution, and 5 mL of 72% sulfuric acid were mixed in an ice-water bath. The mixture was then heated at 60 °C for 10 min and cooled in ice water. Absorbance was measured at 544 nm against a reagent blank, and the results were expressed as mg saponin per gram of extract based on a standard curve of saponin in 80% aqueous methanol.

#### 2.6.3. Condensed Tannins

Condensed tannins were quantified using the vanillin assay, as described by Krause et al. (2023) [32]. The extracts were prepared by mixing 200 mg of the ingredient with 10 mL of absolute methanol for 20 min in rotating screw-cap culture tubes. The supernatant was collected by centrifugation (SL16R centrifuge, Thermo Fischer Scientific, Waltham, MA, USA) at 2740 rcf for 10 min, and 1 mL of the ingredients and catechin standards were transferred in duplicate to glass tubes and heated to 30 °C in a water bath. Simultaneously, the vanillin reagent was prepared by mixing equal parts of 1% (*w*/*v*) vanillin in absolute methanol and 8% (*v*/*v*) concentrated HCl in absolute methanol. Next, 5 mL of the preheated vanillin reagent was added to one set of ingredients and standard tubes, while 5 mL of a preheated 4% aqueous HCl solution was added to the second set of tubes, with a 1 min interval between additions. The ingredients were incubated at 30 °C for 20 min, and the absorbance values were measured by a UV-1800 spectrophotometer (Shimadzu, Kyoto, Japan) at 500 nm at 1 min intervals. Condensed tannins were expressed as catechin equivalents (CE) mg/g, calculated from the standard curve obtained by plotting the absorbance at 500 nm against the reagent blanks.

#### 2.6.4. Trypsin Inhibitor Activity (TIA)

Trypsin inhibitor activity (TIA) was assessed using a spectrophotometric assay following the AOCS Method Ba 12a-2020 33]. One gram of the sample was extracted with 10 mL of a 0.15 M phosphate buffer pH 8.1 at 4 °C overnight. Extracts (200 µL) were incubated with 250 µL of a trypsin solution (0.004% trypsin in 0.025 M glycine HCl buffer) and diluted to 1 mL with at phosphate buffer of pH 8.1. Then 2.5 mL of a 0.001 M BAPNA solution (dissolved in a minimum volume of DMSO with a phosphate buffer, pH 8.1), previously warmed to 37 °C, was added. After incubating the mixture at 37 °C for 15 min in a shaking water bath, the reaction was stopped by adding 300 µL of 30% (*v*/*v*) glacial acetic acid. In this method, one trypsin unit was defined as a 0.02 unit increase in absorbance measured by the UV-1800 spectrophotometer (Shimadzu, Kyoto, Japan) at 410 nm under the 5 mL assay conditions outlined in the protocol [33]. TIA is expressed as trypsin inhibitor units per mg of dry matter.

#### 2.6.5. Chymotrypsin Inhibitor Activity (CIA)

Chymotrypsin inhibitor activity (CIA) was evaluated by following the spectrophotometric method outlined by Alonso et al. (2000) [34]. Ingredients were extracted by mixing the ingredients with 0.05 M Tris-HCl buffer (pH 7.6) overnight at a ratio of 1:10 (ingredient–buffer) (*w*/*v*). Next, 50 µL of the extracts were combined with a 0.005% chymotrypsin solution prepared in 0.05 M Tris-HCl buffer (pH 7.6) (100 μL), followed by dilution to 1 mL. Subsequently, 2.5 mL of 0.001 M benzoyl-L-tyrosine ethyl ester (BTEE), heated to 30 °C, was added to the ingredients, and the absorbance values were immediately recorded at 256 nm by the UV-1800 spectrophotometer (Shimadzu, Kyoto, Japan). In this assay, one chymotrypsin unit corresponded to a 0.01 unit increase in absorbance of the reaction mixture.

### 2.7. Microscopy

Microscopy was performed using a scanning electron microscope (SEM), according to the method reported by Atzler et al. (2021) [35].

### 2.8. Olfactometry

Olfactometry analyses were conducted externally by AromaLAB GmbH (Martinsried, Germany), as described by Gautheron et al. (2024) [21].

### 2.9. Fungal Metabolites

Organic acids were determined by MS-Omics Aps (Vedbæk, Denmark). The ingredients underwent derivatisation with methyl chloroformate, following a slightly modified version of the protocol outlined by Smart et al. (2010) [27], as reported by Gautheron et al. (2024) [21]. Ergosterol concentrations were determined according to the method described by Bickel-Haase et al. (2024) [36].

### 2.10. Statistical Analysis

All analyses were performed in triplicate unless otherwise specified. The obtained results were assessed for normality, followed by a one-way ANOVA with a post hoc Tukey test (*p* < 0.05) was conducted using IBM SPSS Statistics software, version 28 (Armonk, NY, USA). In cases where equal variances were not assumed, correction using the Welch test and Games Howell post hoc test (*p* < 0.05) was applied. Non-normally distributed data were analysed using Kruskal–Wallis tests (*p* < 0.05).

Microsoft Excel Version 2407 (Microsoft Corporation, Redmond, WA, USA) was used to perform correlation analysis and regressions.

## 3. Results

### 3.1. Composition of Ingredients 

The composition of FB, FBA, and FBR are displayed in Table 1. FB had a moisture content of 12.06 g/100 g, while a decrease was observed in FBA and FBR due to the drying operation during the SSF process. FB had a protein content of 24.58%, which increased by 20.3% and 8.1% in FBA and FBR, respectively. The fat content in FB was 2.27% in total, derived mainly from linoleic acid (39.2%), oleic acid (23.3%), and palmitic acid (12.8%). Stearic acid and linolenic acid also contributed to the total fat, with 3.1% and 2.2%, respectively. The complete fatty acid profile can be found in the supporting information (cf. Appendix A, Table A1). With the application of fermentation, the fat content was higher in FBR compared with FBA. The fatty acid profile showed an increase in palmitic acid, oleic acid, and linoleic acid for both genera. However, fermentation with *R. oligosporus* led to a further increase in stearic acid (+200%) and linolenic acid (+140%) compared with FB. Regarding carbohydrates, FB displayed a mono-/disaccharide concentration of 2.21%, derived solely from sucrose, and a total oligosaccharide concentration comprising mainly verbascose (3/4) and raffinose/stachyose (1/4). Total starch showed a balance between digestible starches (51.4%) and resistant starches (48.6%). Finally, total dietary fibre represented 27.88%, mainly from the insoluble fraction (92.5%). After SSF, a reduction in total mono-/disaccharides was observed in FBA, while it increased in FBR. Sucrose was entirely consumed by *A. oryzae*, whereas it was reduced only by a quarter by *R. oligosporus*. An increase in glucose, maltose, and galactose was observed in both fermented ingredients, while fructose production was also noted in the FBR. Oligosaccharides decreased more significantly in FBA, with the values of raffinose/stachyose and verbascose reduced by 94.8% and 95.3%, respectively. In comparison, raffinose/stachyose remained constant in FBR, and verbascose decreased by 77.0%. Small quantities of kestose and nystose were detected in both ingredients. The SSF process led to a reduction in resistant starch by two-thirds and three-quarters for FBA and FBR, respectively. Digestible starch showed an increase of 18.0% in FBA. Finally, fermentation resulted in a decrease in the total dietary fibre content, with a significant reduction in the insoluble fraction (−38% in FBA and −36% in FBR). Both soluble dietary fibre fractions increased in the fermented ingredients.

The nitrogen-to-protein conversion factors for FB, FBA, and FBR are shown in Table 2. FB displayed a conversion factor of 4.97, while fermentation with *A. oryzae* increased it and fermentation with *R. oligosporus* decreased it.

### 3.2. FODMAP Analysis

The FODMAP contents of the ingredients are given in Table 3. Fermentation with *R. oligosporus* resulted in no change in polyols, while an increase of 1.82 g/100 g (arabitol and mannitol) was detected in FBA. Compared with FB, the oligosaccharide content of FBA decreased by almost 18-fold, while the oligosaccharide content in FBR decreased to a lesser extent, by approximately 40%. Overall, the two fermented ingredients showed a reduction in total FODMAPs, with a drop of 45.6% (FBA) and 56.1% (FBR).

### 3.3. Amino Acid Profile

Table 4 displays the total amino acid profile, and their percentages in relation to the 2007 WHO/FAO recommendation for adults and children aged >3 years old [37]. Fermentation with *A. oryzae* or *R. oligosporus* resulted in an increase in most essential amino acids, except for isoleucine, leucine, and threonine. The largest increases observed in FBR were for methionine (+147.6%), cysteine (+70.0%), and histidine (+69.5%). In FBA, the valine concentration increased by 31.8%. FB provided >100% of all essential amino acids except the sulfur amino acids (SAA) (83% of requirements), but fermentation resulted in FBA and FBR providing 132% and 164% of the SAA requirements, respectively. Regarding nonessential amino acids, larger reductions were observed, mainly for arginine, which decreased by 21.5% in FBA and 31.4% in FBR. Although the free amino acids (Table 5) showed higher concentrations after fermentation with *A. oryzae*, most of them increased after fermentation with both fungi, with the exception of asparagine (FBA and FBR), aspartic acid (FBR), and glutamic acid (FBR).

### 3.4. Techno-Functional Properties

The techno-functional properties of FB, FBA, and FBR are displayed in Table 6.

#### 3.4.1. pH and TTA

The pH and TTA are presented in Table 6. FB exhibited a pH value of 6.65 and a TTA of 10.98 mL/10 g. Fermentation resulted in a decrease in pH of 0.39 (FBA) and 0.43% (FBR). The TTA increased significantly by 121.9% (FBA) and 142.3% (FBR).

#### 3.4.2. Sulfhydryl (SH) Groups

Exposed, free, and total SH groups are presented in Table 6. FB showed values of 4.78 μmol SH/g protein (exposed), 5.97 μmol SH/g protein (free), and 101.13 μmol SH/g protein (total). A significant change in the concentration of exposed SH groups was observed after fermentation, decreasing by 23% and 86% in FBA and FBR, respectively. In contrast, fermentation resulted in no significant changes in the concentration of free SH groups, with values of 5.55–6.10 µmol SH/g protein determined in FB, FBA, and FBR. Compared with FB, the total SH groups were significantly lower in FBR (−30.2%), whereas no significant difference was observed after fermentation with *A. oryzae*.

#### 3.4.3. Water- and Oil -Holding Capacity

The water and oil-holding capacity values are shown in Table 6. FB had a water-holding capacity (WHC) of 64.77% and an oil-holding capacity (OHC) of 78.42%. Fermentation increased the WHC of FBA and FBR by 19.0% and 16.8%, respectively. The OHC values determined for FBA and FBR were not significantly different from those of FB; however, FBR had a significantly higher OHC (79.42%) than FBA (75.64%).

#### 3.4.4. Foaming Properties

The foaming properties of the ingredients are given in Table 6. FB showed a foaming capacity of 19.73% and a foam stability of 93.64%. The foaming capacity was reduced by 8.46% (FBA) and 9.92% (FBR) by the SSF process. The foam stability increased to its maximum with *A. oryzae*, while it decreased significantly by 85.2% with *R. oligosporus*.

#### 3.4.5. Minimum Gelling Concentration

The minimum gelling concentration for the three ingredients is presented in Table 6. FB required 24 g/100 g to produce a gel, while fermentation reduced this value to 18 g/100 g and 17 g/100 g for FBA and FBR, respectively.

#### 3.4.6. Protein Solubility

The protein solubility of the ingredients is presented in Table 6. FB showed a protein solubility of 92–100% at its native pH and pH 7. Fermentation significantly reduced the protein solubility at all pH values. At native pH, the protein solubility of FBA (85.06%) was slightly lower than that of FB, with this further decreasing to 50.67% at pH 7. Even more significant reductions in protein solubility were observed after fermentation with *R. oligosporus*, with solubilities of 32.48% and 25.55% determined for FBR at the native pH and pH 7, respectively.

#### 3.4.7. Emulsifying Characteristics

**Figure 1:**
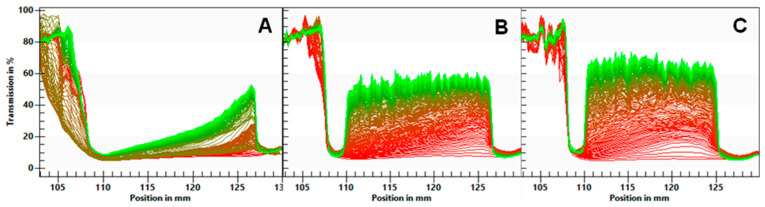
Light transmission profiles of emulsions of fava bean flour (**A**), fava bean flour fermented with *Aspergillus oryzae* (**B**), and fava bean flour fermented with *Rhizopus oligosporus* (**C**) as a function of the position. The left side of each graph represents the top of the ingredient’s cuvette. Red and green lines indicate the initial and latest transmission profiles, respectively.

The separation rates of the ingredients are given in Table 6. The transmission profiles are shown in Figure 1. The FB emulsion showed the slowest phase separation (0.016%), with a gradual increase in transmission. For the FBA and FBR emulsions, the almost immediate increase in the transmission of light along the ingredients’ cuvettes and the formation of a cream layer (visible on the left side of the profile), and a sediment layer (visible on the right side of the profile) show evidence of quicker separation (0.028–0.030%/s).

#### 3.4.8. Particle Size

**Figure A1:**
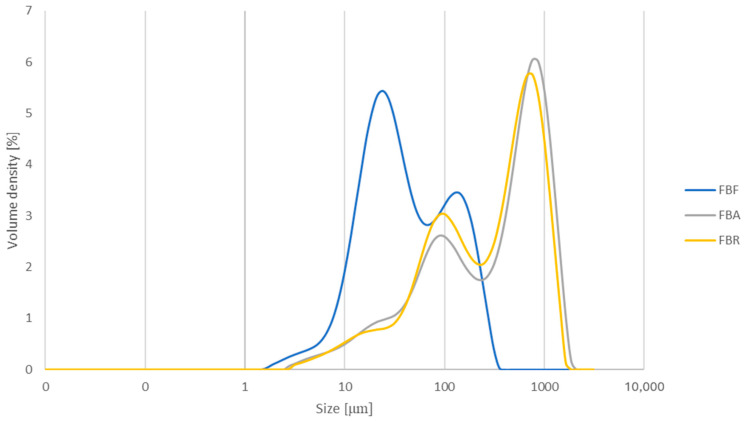
Particle size distribution for fava bean flour (FB), fava bean flour fermented by *Aspergillus oryzae* (FBA), and fava bean flour fermented by *Rhizopus oligosporus* (FBR). The results are expressed as the volume density (%) as a function of size (μm).

The particle size characteristics are provided in Table 6. FB had the smallest particle size, with an average D [4,3] value of 67.67 μm, while the fermented ingredients had larger particle sizes, with 516.00 μm (FBA) and 439.33 μm (FBR). The three particle size percentiles of FB also showed significantly lower values after the SSF process. The highest Dv (10) value was determined for FBR, while FBA presented the highest Dv (50) and Dv (90) values. Furthermore, the particle size distributions of the three ingredients exhibited a bimodal behaviour (see Appendix B, Figure A1).

#### 3.4.9. Colour

The L*, a*, and b* colour values are listed in Table 6. FB showed an L* value of 90.92, an a* value of −1.44, and a b* value of 16.59. SSF reduced the lightness of the ingredients by 17.7% (FBA) and 26.6% (FBR), while slight increases in the a* values and in b* values were observed, indicating more intense red and yellow tones. Overall, FBR showed a higher differential colour index than FBA, indicating a more significant colour difference compared with the unfermented fava bean flour.

#### 3.4.10. Protein Profile

**Figure 2:**
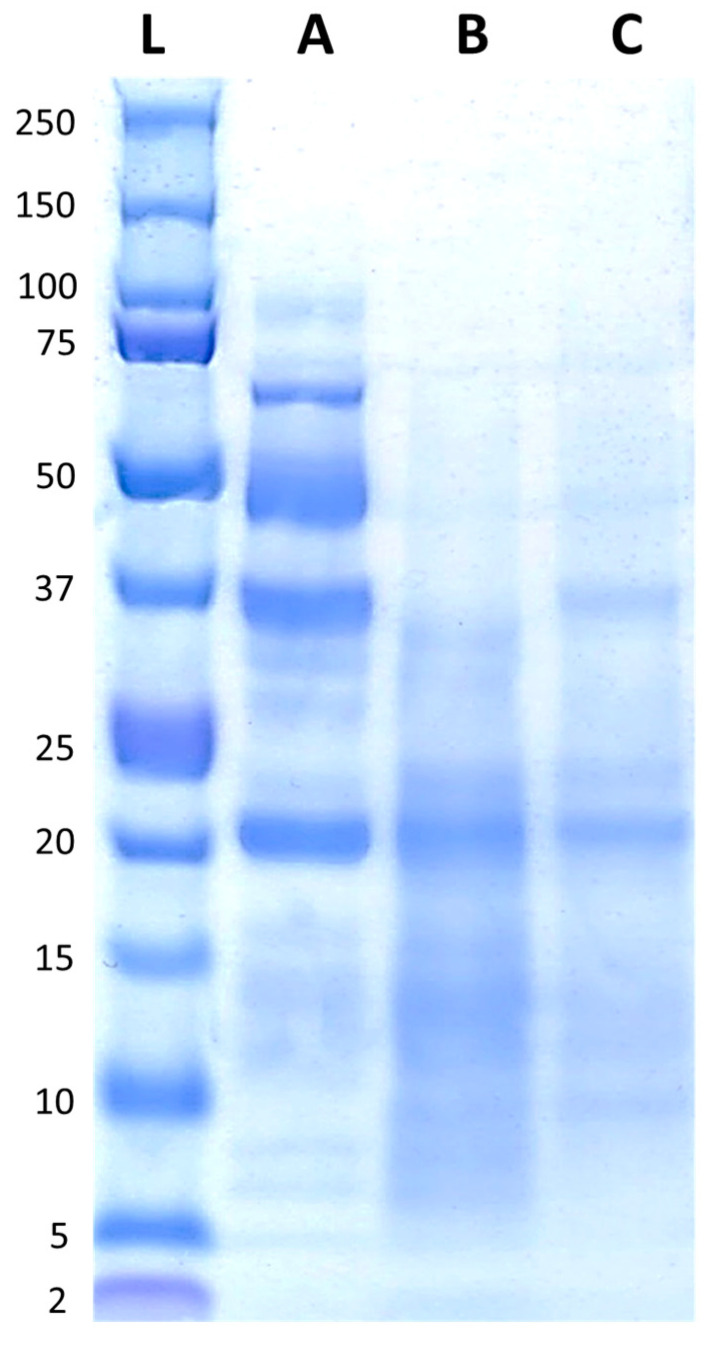
Protein profiles of the fava bean ingredients, with the reference ladder in the first position (L), followed by fava bean flour FB (A), fava bean flour fermented with *Aspergillus oryzae* (B), and fava bean flour fermented with *Rhizopus oligosporus* (C).

The protein profiles of FB, FBA, and FBR are presented in Figure 2. FB displayed peptides with molecular weights ranging from ~5 kDa to ~100 kDa, with bands predominantly visible around ~20 kDa, ~37 kDa, ~50 kDa, and just below 75 kDa. After fermentation, most of the bands decreased in intensity, and were visible between ~5 kDa and ~37 kDa, with a more pronounced band at ~20 kDa.

### 3.5. Microscopy

**Figure 3:**
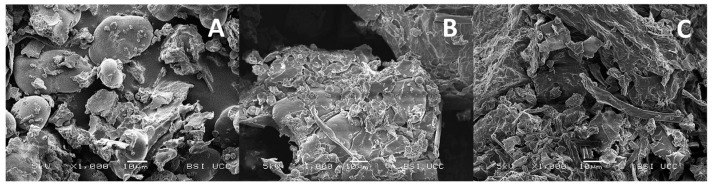
Representative scanning electron micrographs of fava bean flour (**A**), fava bean flour fermented with *Aspergillus oryzae* (**B**), and fava bean flour fermented with *Rhizopus oligosporus* (**C**). The magnification shown is 1000×.

Figure 3 illustrates the SEM micrographs of the ingredients. The differences in morphology between FB and the fermented ingredients (FBA and FBR) are notable. FB contained greater numbers of round and “free” particles. The network of mycelia was observable in the FBA and FBR, resulting in a more compact structure and a rougher surface, which also reflects the larger particle size of the ingredients.

### 3.6. Antinutrients

The antinutrient composition of the ingredients is shown in Table 7. The phytic acid levels in FB (0.397 g/100 g) decreased to a higher extent in FBR (0.214 g/100 g) than in FBA (0.329 g/100 g), with a similar trend observed for chymotrypsin inhibitor concentrations. Condensed tannins in FB were eliminated during fermentation by the two genera. The saponin level in FB was eliminated with *A. oryzae*, while fermentation with *R. oligosporus* reduced saponins by only 19.4%.

### 3.7. Olfactometry

**Figure 4:**
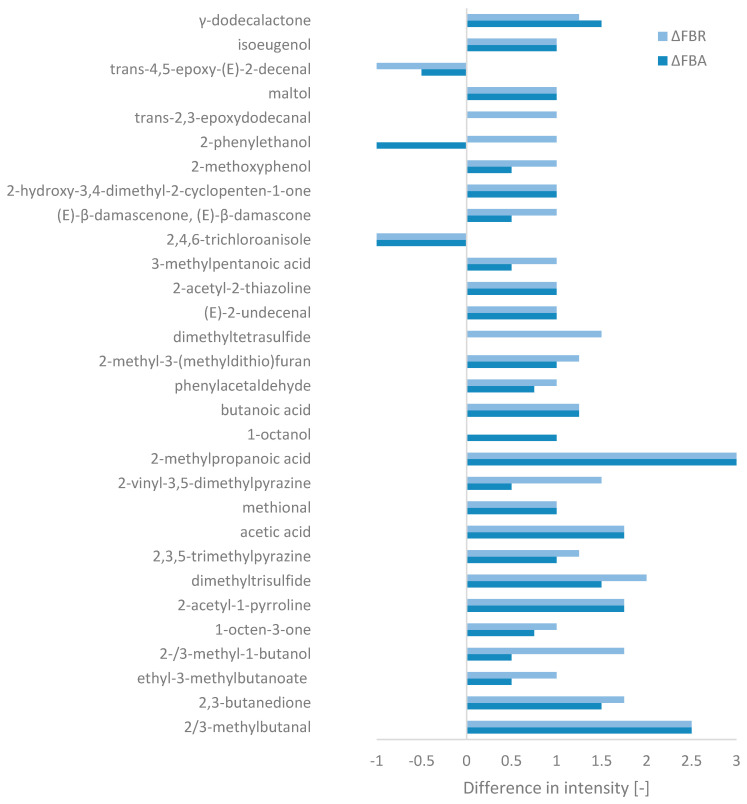
Changes in aromas’ intensity during fermentation relative to fava bean flour, based on the GC-FID peak area (ΔFBA = FBA-FB and ΔFBR = FBR-FB) for compounds with a significant difference (of at least 1 for one of the fermented ingredients).

**Figure A2:**
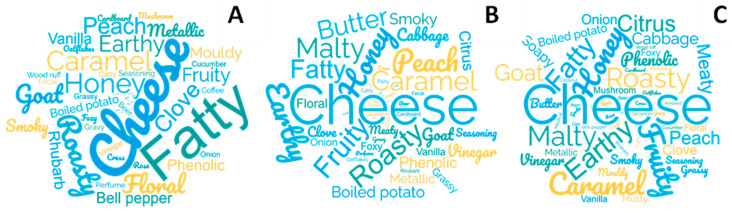
Word clouds for FB (**A**), FBA (**B**), and FBR (**C**) generated from the aroma profiles detected by olfactometry. Larger font sizes reflect the predominance of aromas associated with the compounds.

Olfactometric analysis identified a total of 95 odour-active compounds, including acids, alcohols, aldehydes, ketones, and esters/lactones, as well as one unknown compound (cf. Appendix C, Table A2). Only compounds with significant differences in intensity between FB and FBA/FBR were considered (Figure 4). In addition, Word clouds (cf. Appendix C, Figure A2) were created for each ingredient, based on the aromas associated with the compounds detected (which may be identical).

Among the compounds selected, FB mainly contained trans-4,5-epoxy-(E)-2-decenal (metallic), methional (boiled potato), and acetic acid (vinegar). An unpleasant chemical compound characterised by a mouldy aroma, 2,4,6-trichloroanisole, was also detected. In addition, other aroma-active compounds more characteristic of “savoury” aromas were also present, but with slightly less intensity, such as 2-methoxyphenol (smoky), 2-acetyl-1-pyrroline (roasty), and butanoic acid (cheese). On the other hand, “sweeter” aroma-associated compounds were also detected, such as 2-phenylethanol (honey), ethyl-3-methylbutanoate (fruity), phenylacetaldehyde (honey), and maltol (caramel). The intensity of the abovementioned savoury and sweet aroma compounds were enhanced by fermentation, while the compounds associated with the metallic and mouldy aromas decreased. SSF also led to the formation of new compounds in FBA and FBR, which, with the exception of γ-dodecalactone (peach aroma), relatively strengthened the savoury profile of the raw material. These compounds included 2/3-methylbutanal (malty), 2,3-butanedione (butter), 2-methylpropanoic acid (cheese), 2-methyl-3-(methyldithio)furan (meaty), and 3-methylpentanoic acid (cheese). Sufur compounds such as dimethyltrisulfide and dimethyltetrasulfide (only in FBR) also introduced a cabbage-like aroma to the aromatic profile of the fermented products. An earthy characteristic also appeared due to the presence of certain pyrazine compounds. Overall, in terms of intensity, the biggest changes seemed to occurr with *R. oligosporus*.

### 3.8. Fungal Metabolites

**Figure 5:**
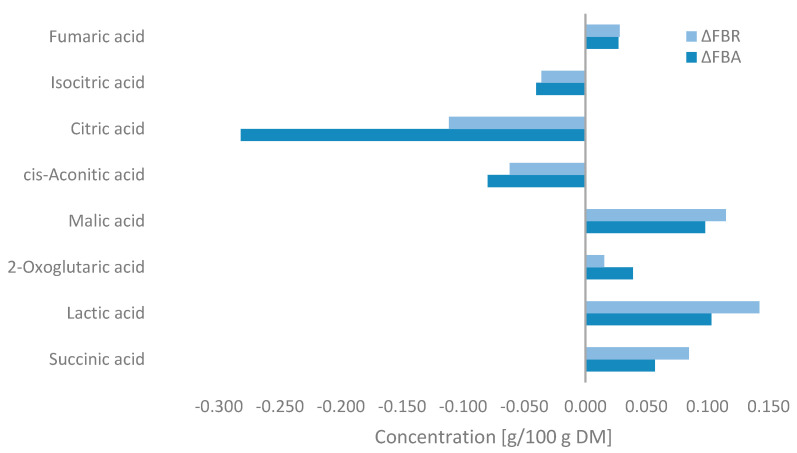
Changes in the organic acid concentrations of fava bean flour fermented with *Aspergillus oryzae* (FBA) or *Rhizopus oligosporus* (FBR) compared with fava bean flour (FB) (ΔFBA = FBA-FB and ΔFBR = FBR-FB) detected by MS-Omics and expressed as g/100 g on a dry matter basis.

Changes in the concentration of organic acids during fermentation are shown in Figure 5. Fermentation resulted in significant decreases in the concentrations of citric acid, cis-aconitic acid, and isocitric acid, with the most notable reduction observed after fermentation with *A. oryzae*. In contrast, lactic acid, malic acid, and succinic acid predominantly increased in FBA and FBR after SSF, with small increases in fumaric acid and 2-oxoglutaric acid levels also being observed. Higher concentrations were observed in FBR.

The ergosterol concentrations of the fermented ingredients were measured after SSF, as an indicator of fungal biomass, and are presented in Table 8. The results showed a slightly higher value with *A. oryzae* compared with *R. oligosporus*.

## 4. Discussion

*Aspergillus oryzae* and *Rhizopus oligosporus* are general recognised as safe (GRAS) fungi that have been used in the food industry for decades [18,19]. Fungal solid-state fermentation (SSF) has been shown to enhance nutritional properties by reducing antinutritional factors, and impacting the techno-functional properties, composition, and sensory characteristics of legumes. In this study, fava bean flour was fermented with *A. oryzae* and *R. oligosporus*, and the effect on the techno-functionality, nutritional profile, and aroma characteristics of the resulting ingredients was investigated.

FODMAPs, an abbreviation for fermentable oligo-, di-, mono-saccharides, and polyols, are a broad category of small nondigestible carbohydrates made up of 1–10 sugar molecules that the small intestine has difficulty absorbing [38]. The decreased sucrose levels, especially in FBA, indicated its possible utilisation during glycolysis. In carbohydrate metabolism, sucrose is first converted to fructose and glucose, which is transformed into D-glucose-6-phosphate. This molecule can then either be converted into D-fructose-6-phosphate further undergoing glycolysis or enter the pentose phosphate pathway (PPP) [39,40]. The PPP is the main source of NADPH, playing a crucial role in fungi by aiding in the production of various important compounds, including polyols, biofuels, carotenoids, and antibiotics [41]. Zaveri et al. (2022) stated that some *Rhizopus* species and strains metabolise sucrose less efficiently than glucose, which would explain the differences in total mono-/disaccharides after SSF between the two genera [42]. Polyols can also have effects such as bloating, pain, changes in bowel habits, and a laxative effect, particularly in people suffering from irritable bowel syndrome (IBS). These effects are due to the malabsorption of these sugar-alcohols and their rapid fermentation by bacteria in the colon, resulting in the production of gas [43]. The complete degradation of sucrose by *A. oryzae* was likely used for polyol production, with a significant negative correlation observed between sucrose and the polyol content (*p*-value: 0.15, r-value: 0.97). According to Kordowska-Wiater (2015), glucose is one of the most efficient precursors for the production of arabitol [44]. As well as the naturally occurring mannitol present in *A. oryzae* cells, an increase in polyols may also be due to the low water activity (a_w_) during SSF, with a low a_w_ resulting in osmotic stress and an accumulation of solutes such as ions, polyols, or amino acids to prevent cellular water loss [45]. Despite the increase in arabitol and mannitol in FBA, the concentration of galacto-oligosaccharides (GOS) was almost completely reduced, resulting in a decrease in total FODMAPs. FBR had a similar FODMAP content to FBA, but *R. oligosporus* reduced the GOS to a lesser extent, with raffinose/stachyose levels remaining unchanged. In accordance with the literature, pulses are considered high in FODMAPs because of their high content of GOS [46].

Fungal amylases are responsible for breaking down starch into the simple sugars glucose and maltose [15,47]. Total starch showed a significant reduction in both fermented ingredients, and this could also be observed in the micrographs. Indeed, the smooth, round, and irregular molecules observed in FB were characteristic of starch molecules [48,49]. These molecules were less numerous in the fermented ingredients, particularly in the FBR, which was also reflected in the lower value of total starch. However, the significant reduction in resistant starch (RS), a type of starch which is resistant or less susceptible to enzymatic hydrolysis, must have occurred in another way, with studies showing that mechanical and physical processes such as grinding and autoclaving could make RS less resistant and more accessible for hydrolysis [50,51]. Pulses’ amyloses can also form part of a complexation with lipids and thus contribute to the RS content [50,52]. These complexes may have been hydrolysed by fungal enzymes, which would explain the increase in digestible starch in the fermented ingredients [53]. Total dietary fibre (TDF) in the FB showed higher values than in previous studies. Millar et al. (2019) reported a total dietary fibre content of 13.8%, of which the insoluble fraction accounted for two-thirds of this value [54]. Resistant starch is considered a form of dietary fibre, which may explain why insoluble dietary fibre accounted for such a large proportion (92.5%) of the TDF in FB [55]. Additionally, Jeraci et al. (1990) stated that the AOAC method for measuring total fibre can be influenced by the presence of certain components such as ash, proteins, tannins, and resistant starches [56].

The production of fatty acids is part of the general metabolic pathway of fungi, through the release of lipases to hydrolyse lipids [57,58]. The increased content of medium-chain fatty acids could also be due to the breakdown of the aforementioned amylose–lipid complexes. FB contained mostly linoleic acid, an essential fatty acid [59], which is the predominant fatty acid in pulses [60]. During SSF, both fermented ingredients showed metabolisation of linoleic acid and oleic acid (a monounsaturated fatty acid with health benefits [61]), as well as palmitic acid (a saturated fatty acid). Additionally, *R. oligosporus* produced stearic acid (a saturated fatty acid) and linolenic acid (an essential fatty acid). Saturated fatty acids are generally reduced in food due to their negative effects on cardiovascular diseases [62]. These changes in the fatty acid composition might be attributed to the increased fat content in the substrate, which acted as an inducing agent for fungal metabolism, explaining the higher total fat content in FBR [19].

Proteases produced by filamentous fungi are responsible for hydrolysing complex proteins into shorter peptides or their constituent amino acids [63]. This was demonstrated in the protein profiles of FBA and FBR determined by SDS-PAGE, which showed a decrease in the molecular weight of protein, reflecting changes in the amino acid composition. An important decrease in arginine, an essential precursor for synthesising compounds such as urea, nitric oxide, and glutamate, as well as other amino acids such as proline [64,65], was also observed. In addition, arginine, which is a basic amino acid, may have decreased due to its destabilisation by the acidic fermentation conditions [66]. Regarding essential amino acids, an increase in the levels of sulfur amino acids was observed after SSF, resulting in complete fulfilment of the daily requirements of adults outlined by the WHO [37]. Filamentous fungi are capable of synthesising cysteine and methionine from serine, with serine being reduced during fermentation in the current study and having a significant negative correlation with cysteine and methionine (cysteine: *p*-value: 0.14, r-value: 0.98; methionine: *p*-value: 0.19, r-value: 0.96) [67,68]. Additionally, some amino acids may have increased due to the breakdown of condensed tannins and insoluble protein complexes by microbial tannase enzymes during fermentation [19]. While condensed tannins were fully eliminated in both fermented ingredients, *A. oryzae* and *R. oligosporus* showed different trends with regards to the degradation of antinutrients in the fava bean substrate. Although *A. oryzae* is known to secrete a large amount of phytase enzymes which are responsible for the degradation of phytic acid, the reduction in phytic acid was low, while *R. oligosporus* showed a higher degree of degradation. A similar trend was previously observed after SSF of a quinoa substrate with *A. orzyzae* and *R. oligosporus* [21]. This could also explain the decreased level of starch degradation observed in FBA, as phytic acid can bind starch [6]. As also observed in a separate study [21], *A. oryzae* reduced chymotrypsin inhibitors to a lower level than *R. oligosporus*. However, saponins were fully eliminated in FBA, whereas only a slight reduction was observed in FBR. Saponins are commonly present in pulses and contribute a bitter taste that may limit consumer acceptability [69]. Furthermore, the formation of trypsin inhibitors may hinder the absorption of dietary proteins, with these compounds capable of binding to the active sites of pancreatic trypsin, resulting in a reduction in the enzyme’s proteolytic activity [70].

The protein solubility of both ingredients decreased after SSF. Since a lower protein solubility was observed in FBR, as well as a higher concentration of trypsin inhibitors, this may be a reason. Indeed, a negative correlation occurred between trypsin inhibitors and protein solubility (protein solubility at pH 7: *p*-value: 0.04, r-value: 0.998; protein solubility at native pH: *p*-value: 0.33, r-value: 0.87). However, protein solubility may also have been affected by other factors (intrinsic and extrinsic), such as the observed increase in the protein content of the fermented ingredients. Changes in the amino acid composition and the conformation of proteins have an impact on proteins’ solubility [71,72]. Because of the ability of filamentous fungi to assimilate complex substrates, they produce a protein-rich fungal biomass called mycoproteins [73]. This network of mycelia and porous microstructures was clearly observed in the microscopic images. The aggregated surfaces also resulted in larger particles, which is also an important factor for the reduction in protein solubility [72].

The hydrolysis of proteins by the proteolytic activity of fungi exposes their hydrophobic and/or hydrophobic sites by unfolding the proteins’ structure [74,75], potentially enhancing techno-functional properties such as the gelation, foaming characteristics and emulsifying properties [76]. In this study, a decrease in the foaming capacity of FBA and FBR and the foam stability of FBR was observed. This could potentially be due to the exposure of hydrophobic sites and an increased likelihood of absorption at the air–water interface, thereby reducing the interfacial tension [77]. Moreover, it may also be due to extensive protein denaturation, as well as increased particle size [76,78]. A significant negative correlation between the mean particle size and foaming capacity (*p*-value: 0.17, r-value: 0.96) was observed. Indeed, hydrophobic exposure was also responsible for the formation of aggregates [74], with aggregates in the powder resulting in significant changes in the techno-functional properties. The increase in the emulsions’ separation rates was also positively correlated with the mean particle size (*p*-value: 0.17, r-value: 0.97) [79]. FBR also showed a higher level of sedimentation in an emulsion, which may be a reflection of its lower protein solubility [79]. The formation of aggregates can also result in to the development of structures called microcapillaries, which have internal spaces that can physically trap oil, thus increasing the oil-holding capacity (OHC) [74]. However, in this study, no significant difference in the OHC of fava bean flour was observed after SSF. It is possible that enzymatic hydrolysis may have exposed a more significant amount of hydrophilic binding sites, contributing to the increased water-holding capacity (WHC) of both fermented ingredients [75]. WHC was also found to have a significant negative correlation with the minimum gelling concentration (*p*-value: 0.15, r-value: 0.97). Many studies have linked these two properties, as a high WHC can aid in the binding of water, resulting in a stronger gel structure [80,81].

Colour changes (ΔE) were observed in both FB and FBR after fermentation, with both ingredients showing lower L* values as well as higher a* and b* values [82]. Colour changes may occur during SSF due to fungal growth through the production of mycelia and/or spores [83]. This could also explain the higher differential colour index of FBR, as different fungal species can produce different mycelia, which vary in colour [84]. However, this could also be due to the autoclaving process carried out on the ingredients prior to fermentation [82].

Olfactometric analysis revealed that fermenting fava bean flour with *A. oryzae* and *R. oligosporus* produced 3-methylpentanoic acid, butanoic acid, and 2-methylpropanoic acid, which are associated with cheese aromas, along with acetic acid, which gives a vinegar aroma. Acetic and butanoic acids have pyruvate as a precursor, a product of sugar metabolism, while 2-methylpropanoic and 3-methylpentanoic acids were derived from their respective aldehydes [85]. The main pathway for synthesising aromatic compounds begins with the oxidative deamination of amino acids, producing an α-keto acid, which is decarboxylated to form an aldehyde. The aldehyde can then be oxidised to an acid or reduced to an alcohol [85]. Key amino acids in this process include valine, leucine, isoleucine, methionine, cysteine, phenylalanine, proline, and lysine. Valine, leucine, isoleucine, phenylalanine, and methionine produce aldehydes through the Strecker degradation pathway [86]. In this study, the Strecker reaction produced 2-methylbutanal (malty), 3-methylbutanal (malty), phenylacetaldehyde (honey), and methional (boiled potato) from isoleucine, leucine, phenylalanine, and methionine, respectively [85,86,87]. Methionine also formed the sulfur compounds dimethyltrisulfide, with a higher content in FBR, and dimethyltetrasulfide, found only in FBR, resulting in a cabbage-like aroma [86,88]. Earthy aromas from fermented products are due to pyrazine compounds, which again were slightly more developed in FBR, likely derived from lysine [86]. 2-Acetyl-1-pyrroline produced a roasty aroma from proline [86], and cysteine may be the source of the meaty aroma of 2-methyl-3-(methyldithio)furan [89,90]. Additionally, sweet aromas such as γ-dodecalactone (peach) and maltol (caramel) were also developed in the fermented products. γ-Dodecalactone, this time predominant in FBA, may have been produced through a different pathway where fungi transform certain fatty acids [91], with oleic acid being the likely source [92]. Maltol resulted from the Maillard reaction [93]. SSF also appeared to enhance the aroma profile by reducing 2,4,6-trichloroanisole and trans-4,5-epoxy-(E)-2-decenal, which gave fava bean flour a mouldy and metallic-like aroma.

Some filamentous fungi are of great interest for their high production of organic acids. Lactic acid was the organic acid produced most by both fungi. Glucose undergoes glycolysis to produce pyruvate and ATP for cellular energy. Pyruvate is then converted to pyruvic acid and further into lactic acid by fungi [58,94]. Furthermore, citric acid is typically produced in high amounts during fermentation from two pyruvic acid molecules via the TCA cycle [95]. However, in this study, citric acid levels were significantly lower, possibly due to the duration of fermentation. Normally, one pyruvic acid molecule becomes acetyl-CoA, and the other becomes oxaloacetic acid, which enters the mitochondria and converts to malic acid, then citric acid with acetyl-CoA [96]. The significant production of malic acid suggests incomplete fermentation. Fumaric and succinic acids are intermediates of the TCA cycle, and fungi are also capable of using a reducing TCA cycle in the cytosol, converting pyruvic acid to malic acid, oxaloacetic acid, fumaric acid, and finally succinic acid [96]. This would explain the increase in succinic and fumaric acids. All these acids showed a strong positive correlation with glucose (lactic acid: *p*-value 0.09, r-value 0.989; malic acid: *p*-value 0.01, r-value 1.00; fumaric acid: *p*-value 0.06, r-value 0.996; succinic acid: *p*-value 0.13, r-value 0.979). The drop in pH and rise in TTA values during SSF were linked to the fungi’s acid production.

## 5. Conclusions

SSF with the filamentous fungi *A. oryzae* and *R. oligosporus* resulted in changes in the nutritional, functional, and aromatic profile of fava bean flour. An increase in the protein and fat content were observed in the fermented ingredients, while levels of starch, fibre, and oligosaccharides generally decreased. The nutritional quality of FB was improved, fulfilling the WHO’s recommended daily amino acid requirements for all essential amino acids, with a reduction in most antinutrients observed. In addition, the fungi produced acids during their metabolism, with a sharp increase in concentrations of malic, lactic, and succinic acids, while a significant decrease in citric acid was observed. In terms of the techno-functional properties, the WHC of FBA and FBR increased, as did the foam stability for FBA. On the other hand, the OHC, minimum gelling concentrations, and foaming properties decreased. After the SSF process, the emulsions separated more rapidly, and an increase in particle size was observed. SSF modulated the aroma profile, mainly intensifying compounds associated with savoury aromas such as cheese, malty, cabbage, vinegar, roasty, and butter-like aromas, although some sweeter aromas, such as peach and caramel, were also identified. Overall, the aroma changes were more intense in the FBR, with SSF aiding in the reduction of the flour’s metallic and mouldy aromas. In a comparison of the two fermented products, FBA may offer a superior nutritional profile, with a higher protein content, lower fat and sugar contents, higher protein solubility, higher foam stability, and less significant colour changes. However, fermentation with *R. oligosporus* could be more effective for the production of desirable aroma-associated compounds and organic acids, while also increasing dietary fibre and reducing FODMAP contents. In summary, both ingredients produced by SSF of fava bean flour with *A. oryzae* and *R. oligosporus* present interesting nutritional, functional, and aroma characteristics. Future research should focus on the investigation of their sensory properties, food applications, and consumer acceptance.

## Figures and Tables

**Table 1:** Composition of fava bean flour (FB), fava bean flour fermented by *Aspergillus oryzae* (FBA), and fava bean flour fermented by *Rhizopus oligosporus* (FBR). The results are expressed on a dry matter basis, in g/100 g ± standard deviation. n.d. indicates not detected.

	g/100 g		
	FB	FBA	FBR
**Moisture**	12.06 ± 0.09	8.37 ± 0.13	7.54 ± 0.03
	g/100 g DM		
**Protein**	24.58 ± 0.18	29.56 ± 0.06	26.57 ± 0.18
Total Nitrogen	4.95 ± 0.22	5.78 ± 0.20	5.87 ± 0.19
**Total fats**	2.27 ± 0.14	2.65 ± 0.31	3.18 ± 0.37
**Fatty acid profile**			
Palmitic acid	0.29 ± 0.05	0.36 ± 0.06	0.43 ± 0.06
Stearic acid	0.07 ± 0.01	0.06 ± 0.01	0.21 ± 0.05
Oleic acid	0.53 ± 0.07	0.60 ± 0.08	0.79 ± 0.10
Linoleic acid	0.89 ± 0.11	1.05 ± 0.13	0.96 ± 0.12
Linolenic acid	0.05 ± 0.01	0.05 ± 0.01	0.12 ± 0.02
**Carbohydrates**			
**Mono-/** **d** **isaccharides**	2.21 ± 0.08 ^a^	1.67 ± 0.27 ^b^	3.67 ± 0.15 ^c^
Glucose	n.d. ^a^	0.93 ± 0.16 ^b^	1.07 ± 0.03 ^b^
Fructose	n.d. ^a^	n.d. ^a^	0.13 ± 0.01 ^b^
Sucrose	2.21 ± 0.08 ^a^	n.d. ^b^	1.67 ± 0.03 ^c^
Maltose	n.d. ^a^	0.71 ± 0.11 ^b^	0.75 ± 0.07 ^b^
Galactose	n.d. ^a^	0.03 ± 0.00 ^b^	0.06 ± 0.00 ^c^
**Oligosaccharides**	3.74 ± 0.24 ^a^	0.21 ± 0.01 ^b^	1.64 ± 0.05 ^c^
Raffinose/stachyose	0.96 ± 0.05 ^a^	0.05 ± 0.00 ^b^	0.98 ± 0.04 ^a^
**Verbascose**	2.78 ± 0.19 ^a^	0.13 ± 0.00 ^b^	0.64 ± 0.01 ^c^
**Kestose**	n.d. ^a^	0.01 ± 0.00 ^b^	0.01 ± 0.00 ^c^
**Nystose**	n.d. ^a^	0.03 ± 0.00 ^b^	0.01 ± 0.00 ^c^
**Starch**			
Digestible starch	25.69 ± 0.55 ^a^	34.62 ± 1.69 ^b^	28.43 ± 2.12 ^a,b^
Resistant starch	24.31 ± 0.46 ^a^	9.43 ± 0.96 ^b^	6.39 ± 0.37 ^c^
Total starch	50.00 ± 0.87 ^a^	44.04 ± 2.28 ^b^	34.83 ± 2.15 ^c^
**Dietary fibre**			
Soluble low molecular weight (SDFS)	1.17 ± 0.06 ^a^	2.45 ± 0.06 ^b^	3.16 ± 0.13 ^c^
Soluble high molecular weight (SDFP)	0.92 ± 0.35 ^a^	2.18 ± 0.29 ^b^	2.46 ± 0.28 ^b^
Insoluble (IDF)	25.79 ± 0.68 ^a^	15.93 ± 0.22 ^b^	16.45 ± 1.17 ^b^
Total dietary fibre (TDF)	27.88 ± 0.59 ^a^	20.59 ± 0.44 ^b^	22.07 ± 0.34 ^b^
**Ash**	3.57 ± 0.22	3.90 ± 0.26	3.92 ± 0.26

Results within the same row that share the same letter do not differ significantly (*p* < 0.05).

**Table 2:** Nitrogen-to-protein conversion factor for fava bean flour (FB), fava bean flour fermented by *Aspergillus oryzae* (FBA), and fava bean flour fermented by *Rhizopus oligosporus* (FBR), used to calculate protein solubility.

	Kjeldahl Conversion Factor
Fava bean flour (FB)	4.97
Fava bean flour fermented with *Aspergillus oryzae* (FBA)	5.11
Fava bean flour fermented with *Rhizopus oligosporus* (FBR)	4.52

**Table 3:** FODMAP content in fava bean flour (FB), fava bean flour fermented by *Aspergillus oryzae* (FBA), and fava bean flour fermented by *Rhizopus oligosporus* (FBR). The results are expressed on a dry matter basis, in g/100 g ± standard deviation. * EF denotes excess fructose, calculated as glucose–fructose. n.d. indicates not detected.

FODMAP	g/100 g DM		
	FB	FBA	FBR
Excess fructose (EF) *	-	-	-
*Glucose*	n.d. ^a^	0.93 ± 0.16 ^b^	1.07 ± 0.03 ^b^
*Fructose*	n.d. ^a^	n.d.^a^	0.13 ± 0.01 ^b^
∑Polyols	0.01 ± 0.00 ^a^	1.83 ± 0.21 ^b^	0.01 ± 0.00 ^a^
*Arabitol*	n.d. ^a^	1.23 ± 0.14 ^b^	n.d. ^a^
*Sorbitol*	0.01 ± 0.00 ^a^	n.d.^b^	0.01 ± 0.00 ^c^
*Mannitol*	n.d. ^a^	0.60 ± 0.07 ^b^	n.d. ^a^
∑Oligosaccharides	3.74 ± 0.24 ^a^	0.22 ± 0.01 ^b^	1.64 ± 0.05 ^c^
*Raffinose/* *s* *tachyose*	0.96 ± 0.05 ^a^	0.05 ± 0.00 ^b^	0.98 ± 0.04 ^a^
*Verbascose*	2.78 ± 0.19 ^a^	0.13 ± 0.00 ^b^	0.64 ± 0.01 ^c^
*Kestose*	n.d. ^a^	0.01 ± 0.00 ^b^	0.01 ± 0.00 ^c^
*Nystose*	n.d. ^a^	0.03 ± 0.00 ^b^	0.01 ± 0.00 ^c^
Total fructans	-	-	-
∑FODMAPs	3.75 ± 0.24 ^a^	2.04 ± 0.22 ^b^	1.65 ± 0.05 ^b^

Results within the same row that share the same letter do not differ significantly (*p* < 0.05).

**Table 4:** Amino acid profiles of fava bean flour (FB), fava bean flour fermented by *Aspergillus oryzae* (FBA), and fava bean flour fermented by *Rhizopus oligosporus* (FBR). The total amino acids were quantified and expressed as g/100 g protein ± standard deviation, and the daily requirements for each essential amino acid were determined according to the WHO (2007).

	FB		FBA		FBR	
	Level (g/100 g protein) *	% Requirement **	Level (g/100 g protein)	% Requirement	Level (g/100 g protein)	% Requirement
**Essential AA**						
Histidine	2.98 ± 0.42	198.92	3.52 ± 0.15	234.65	5.05 ± 0.42	336.81
Isoleucine	5.26 ± 0.74	175.48	5.23 ± 0.18	174.35	5.05 ± 0.66	168.29
Leucine	9.74 ± 1.36	165.05	9.40 ± 0.36	159.40	8.93 ± 1.16	151.34
Lysine	8.07 ± 1.12	179.39	8.72 ± 0.33	193.80	8.31 ± 1.13	184.72
Methionine	0.63 ± 0.08		1.22 ± 0.05		1.56 ± 0.01	
Cysteine	1.20 ± 0.14		1.68 ± 0.09		2.04 ± 0.03	
Methionine + cysteine	1.83 ± 0.22	83.27	2.90 ± 0.13	131.78	3.61 ± 0.04	163.89
Phenylalanine	5.18 ± 0.72		5.48 ± 0.23		5.50 ± 0.71	
Tyrosine ***	3.25 ± 0.45		3.39 ± 0.19		3.73 ± 0.50	
Phenylalanine + tyrosine	8.42 ± 1.18	221.68	8.87 ± 0.42	233.29	9.23 ± 1.22	242.92
Threonine	5.00 ± 0.70	217.42	4.99 ± 0.20	217.07	4.89 ± 0.62	212.42
Tryptophan	0.60 ± 0.06	100.23	0.93 ± 0.02	154.59	0.95 ± 0.05	158.76
Valine	4.56 ± 0.64	116.96	6.01 ± 0.22	154.22	6.06 ± 0.81	155.33
**Nonessential AA**						
Alanine	5.09 ± 0.71		5.65 ± 0.20		7.49 ± 0.97	
Arginine	11.50 ± 1.60		9.03 ± 0.41		7.89 ± 1.09	
Aspartic acid + asparagine	14.21 ±1.98		13.55 ± 0.49		13.17 ± 1.71	
Glutamic acid + glutamine	21.67 ± 3.02		20.59 ± 0.76		19.51 ± 2.44	
Proline	4.56 ± 0.64		5.05 ± 0.19		5.07 ± 0.61	
Serine	6.84 ± 0.95		6.51 ± 0.26		5.97 ± 0.78	
Glycine	5.53 ± 0.77		5.23 0.18		5.12 ± 0.65	

* Calculated by dividing the amino acid content (g/100 g DM) by the true protein content (sum of amino acid residues). ** Calculated by determining the ratio of each essential amino acid per 100 g of protein to the requirement specified by the WHO (2007). *** Tyrosine, although not an essential amino acid, is included because its requirement is combined with that of phenylalanine.

**Table 5:** Free amino acid content of fava bean flour (FB), fava bean flour fermented by *Aspergillus oryzae* (FBA), and fava bean flour fermented by *Rhizopus oligosporus* (FBR). The results are expressed on a dry matter basis in g/100 g ingredient ± standard deviation.

	g/100 g Ingredient DM		
Free AA	FB	FBA	FBR
Alanine	0.044 ± 0.002	0.180 ± 0.002	0.186 ± 0.008
Asparagine	0.041 ± 0.017	0.026 ± 0.002	0.028 ± 0.002
Aspartic acid	0.126 ± 0.078	0.188 ± 0.003	0.066 ± 0.004
Cysteine	0.000 ± 0.001	0.011 ± 0.001	0.006 ± 0.000
Glutamic acid	0.239 ± 0.108	0.335 ± 0.020	0.206 ± 0.011
Glutamine	0.002 ± 0.002	0.024 ± 0.002	0.026 ± 0.001
Glycine	0.002 ± 0.001	0.008 ± 0.000	0.007 ± 0.000
Histidine	0.004 ± 0.001	0.103 ± 0.009	0.090 ± 0.003
Isoleucine	0.011 ± 0.007	0.135 ± 0.002	0.095 ± 0.007
Leucine	0.009 ± 0.006	0.148 ± 0.003	0.093 ± 0.006
Lysine	0.033 ± 0.016	0.229 ± 0.023	0.133 ± 0.005
Methionine	0.001 ± 0.001	0.021 ± 0.001	0.003 ± 0.000
Ornithine	0.003 ± 0.002	0.018 ± 0.001	0.032 ± 0.001
Phenylalanine	0.046 ± 0.028	0.151 ± 0.003	0.113 ± 0.006
Proline	0.003 ± 0.002	0.018 ± 0.000	0.012 ± 0.001
Serine	0.007 ± 0.003	0.097 ± 0.005	0.053 ± 0.003
Threonine	0.006 ± 0.004	0.089 ± 0.003	0.055 ± 0.004
Tryptophan	0.010 ± 0.007	0.043 ± 0.002	0.022 ± 0.001
Tyrosine	0.015 ± 0.009	0.099 ± 0.004	0.059 ± 0.003
Valine	0.014 ± 0.009	0.106 ± 0.001	0.079 ± 0.006

**Table 6:** Techno-functional properties of fava bean flour (FB), fava bean flour fermented by *Aspergillus oryzae* (FBA), and fava bean flour fermented by *Rhizopus oligosporus* (FBR). The results are presented as mean ± standard deviation.

	FB	FBA	FBR
pH	6.65 ± 0.01 ^a^	6.26 ± 0.03 ^b^	6.22 ± 0.01 ^b^
TTA (mL 0.1 M NaOH/10g ingredient)	10.98 ± 0.03 ^a^	24.37 ± 0.51 ^b^	26.60 ± 0.36 ^c^
SH groups (μmol SH/g protein)			
*Exposed*	4.78 ± 0.30 ^a^	3.67 ± 0.33 ^b^	0.68 ± 0.03 ^c^
*Free*	5.97 ± 0.25 ^a^	5.55 ± 0.91 ^a^	6.10 ± 0.17 ^a^
*Total*	101.13 ± 8.43 ^a^	108.17 ± 9.09 ^a^	70.61 ± 4.70 ^b^
Water-holding capacity (%)	64.77 ± 0.17 ^a^	77.05 ± 1.33 ^b^	75.64 ± 0.71 ^c^
Oil-holding capacity (%)	78.42 ± 0.54 ^a,b^	75.64 ± 0.71 ^a^	79.42 ± 0.74 ^b^
Foaming capacity (%)	19.73 ± 3.12 ^a^	11.27 ± 1.30 ^b^	9.81 ± 2.56 ^b^
Foam stability (%)	93.64 ± 5.53 ^a^	100.00 ± 0.00 ^a^	13.89 ± 2.41 ^b^
Minimum gelling concentration (%)	24.00 ± 0.00 ^a^	18.00 ± 0.00 ^b^	17.00 ± 0.00 ^c^
Protein solubility (%)			
*Native pH*	100.13 ± 2.06 ^a^	50.68 ± 1.77 ^b^	25.55 ± 0.99 ^c^
*pH 7*	92.63 ± 4.59 ^a^	85.06 ± 1.55 ^b^	32.48 ± 0.50 ^c^
Separation rate (%/s)	0.016 ± 0.002 ^a^	0.028 ± 0.001 ^b^	0.030 ± 0.002 ^b^
Particle size distribution (μm)			
*D [4,3]*	67.67 ± 0.81 ^a^	516.00 ± 10.54 ^b^	439.33 ± 2.08 ^c^
*Dv (10)*	12.23 ± 0.06 ^a^	32.63 ± 1.70 ^b^	37.03 ± 0.61 ^b^
*Dv (50)*	36.90 ± 0.26 ^a^	414.00 ± 31.43 ^b^	335.33 ± 16.17 ^b^
*Dv (90)*	174.00 ± 2.65 ^a^	1196.67 ± 20.82 ^b^	1020.00 ± 10.00 ^c^
Colour			
L* value	90.92 ± 0.50 ^a^	74.82 ± 0.15 ^b^	66.72 ± 0.67 ^c^
a* value	−1.44 ± 0.02 ^a^	4.32 ± 0.07 ^b^	4.29 ± 0.07 ^b^
b* value	16.59 ± 0.47 ^a^	21.50 ± 0.00 ^b^	19.98 ± 0.33 ^c^
*Differential colour index ΔE*	-	17.80 ± 0.59	25.11 ± 0.23

Results within the same row that share the same letter do not differ significantly (*p* < 0.05).

**Table 7:** Content of the antinutritional factors in fava bean flour (FB), fava bean flour fermented by *Aspergillus oryzae* (FBA), and fava bean flour fermented by *Rhizopus oligosporus* (FBR). Values are presented on a dry matter basis as the mean ± standard deviation.

	FB	FBA	FBR
Phytic acid (g/100 g)	0.397 ± 0.001	0.329 ± 0.001	0.214 ± 0.002
Saponins (GAE mg/g)	0.31 ± 0.09	0.00 ± 0.00	0.25 ± 0.03
Trypsin inhibitors (TIU/mg)	0.00 ± 0.00	0.82 ± 0.04	4.22 ± 0.14
Chymotrypsin inhibitors (CIU/mg)	36.00 ± 0.28	25.70 ± 0.42	12.20 ± 0.28
Condensed tannins (catechin equivalent mg/g)	1.56 ± 0.98	0.02 ± 0.02	0.00 ± 0.00

**Table 8:** Ergosterol concentrations of fava bean flour (FB), fava bean flour fermented by *Aspergillus oryzae* (FBA), and fava bean flour fermented by *Rhizopus oligosporus* (FBR).

Ingredient	Ergosterol (mg/100 g)
Fava bean flour (FB)	0.0 ± 0.0
Fava bean flour + *Aspergillus oryzae* (FBA)	195.1 ± 2.8
Fava bean flour + *Rhizopus oligosporus* (FBR)	187.6 ± 0.6

## Data Availability

The original contributions presented in the study are included in the article, further inquiries can be directed to the corresponding author.

## Appendix A. Total Fatty Acid Profile

**Table A1:** Total fatty acid profile of fava bean flour (FB), fava bean flour fermented by *Aspergillus oryzae* (FBA), and fava bean flour fermented by *Rhizopus oligosporus* (FBR). The results are expressed on a dry matter basis in g/100 g ± standard deviation. n.d. = not detected.

Fatty Acid Profile		g/100 g DM		
		FB	FBA	FBR
Lauric acid	12:0	0.01 ± 0.00	n.d.	n.d.
Myristic acid	14:0	0.00 ± 0.00	0.01 ± 0.00	0.01 ± 0.00
Pentadecanoic acid	15:0	0.00 ± 0.00	0.01 ± 0.00	0.01 ± 0.00
Palmitic acid	16:0	0.29 ± 0.05	0.36 ± 0.06	0.43 ± 0.06
Hexadecenoic acid	16:1	0.00 ± 0.00	0.02 ± 0.00	0.02 ± 0.00
Isoeptadecanoic acid	17:0	n.d.	n.d.	n.d.
14-Methyl hexadecanoic acid	17:0	n.d.	0.00 ± 0.00	n.d.
Margaric acid	17:0	0.00 ± 0.00	0.00 ± 0.00	0.00 ± 0.00
Heptadecenoic acid	17:1	n.d.	n.d.	0.02 ± 0.00
Stearic acid	18:0	0.07 ± 0.01	0.06 ± 0.01	0.21 ± 0.05
Oleic acid	18:1	0.53 ± 0.07	0.60 ± 0.08	0.79 ± 0.10
Linoleic acid	18:2	0.89 ± 0.11	1.05 ± 0.13	0.96 ± 0.12
Conjugated linoleic acid	18:2	n.d.	n.d.	n.d.
Linolenic acid	18:3	0.05 ± 0.01	0.05 ± 0.01	0.12 ± 0.02
Arachidic acid	20:0	0.02 ± 0.00	0.01 ± 0.00	0.02 ± 0.00
Eicosenoic acid	20:1	0.01 ± 0.00	0.01 ± 0.00	0.01 ± 0.00
Heneicosylic acid	21:0	n.d.	n.d.	n.d.
Behenic acid	22:0	0.00 ± 0.00	n.d.	0.01 ± 0.00
Docosanoic acid	22:1	n.d.	n.d.	n.d.
Lignoceric acid	24:0	n.d.	n.d.	0.01 ± 0.00

## Appendix C. Aroma Profile

**Table A2:** Full aroma profile of fava bean flour (FB), fava bean flour fermented by *Aspergillus oryzae* (FBA), and fava bean flour fermented by *Rhizopus oligosporus* (FBR). The results include the compounds’ names, odour quality, and intensities.

Compound ^1^	Odour Quality ^2^	RI^2^ (FFAP)	Intensity ^3,4^		
			FB	FBA	FBR
Methylpropanal	Malty	<900	0	0.75	0.5
2/3-Methylbutanal	Malty	920	0	2.5	2.5
Methylbutanoate	Fruity	973	0	0.5	0.75
2,3-Butanedione	Butter	987	0	1.5	1.75
Unknown	Onion, garlic	1019	0	0	0.5
Unknown	Gas	1042	0	0	0.5
Ethyl-2-methylbutanoate	Fruity	1050	0.5	1.25	1
Ethyl-3-methylbutanoate	Fruity	1069	0.75	1.25	1.75
Hexanal	Grassy	1088	1	1.5	1.75
3-Methyl-2-buten-1-thiol	Beer	1108	0.5	0.5	1
2-Heptanone	Fruity	1178	0	0	0.5
2-/3-Methyl-1-butanol	Malty	1207	0	0.5	1.75
(Z)-4-heptenal	Fishy	1241	0	0.5	0.5
3-Hydroxy-2-butanone	Butter	1280	0	0.75	0.5
Octanal	Citrus	1285	0	0	0.5
1-Octen-3-one	Mushroom	1295	0.75	1.5	1.75
2-Acetyl-1-pyrroline	Roasty	1329	0.5	2.25	2.25
1-Mercapto-2-propanone	Catty	1363	0.75	0.5	0.5
Dimethyltrisulfide	Cabbage	1366	0	1.5	2
(E)/(Z)-1,5-octadien-3-one	Geranium	1368	0	0	0.75
2,3,5-Trimethylpyrazine	Earthy	1408	0	1	1.25
2-Isopropyl-3-methoxypyrazine	Bell pepper	1425	0.5	0	0
2-Furfurylthiol	Coffee	1428	0.5	0	0
Acetic acid	Vinegar	1433	1.25	3	3
Methional	Boiled potato	1450	1.5	2.5	2.5
2-Ethyl-3,5-dimethylpyrazine	Earthy	1455	1	1.5	1
(Z)-2-nonenal	Fatty	1495	0.5	0	1
2-Isobutyl-3-methoxypyrazine	Bell pepper	1518	0.5	0	0.5
(E)-2-nonenal	Cardboard	1523	0.75	0.5	1
Linalool	Floral	1544	1	0.5	0.5
2-Vinyl-3,5-dimethylpyrazine	Earthy	1551	0	0.5	1.5
2-Methylpropanoic acid	Cheese	1554	0	3	3
1-Octanol	Citrus	1559	0	1	0
2-Acetyl-1,4,5,6-tetrahydropyridine	Roasty	1564	0.5	0	0
(E,Z)-2,6-nonadienal	Cucumber	1574	1	0	0.5
(Z)-2-decenal	Fatty	1603	0.5	0	0
Benzyl mercaptane	Cress	1611	0.5	1	0.75
2-Acetylpyrazine	Roasty	1618	0	0	0.5
Butanoic acid	Cheese	1621	0.5	1.75	1.75
Phenylacetaldehyde	Honey	1632	0.5	1.25	1.5
2-Methyl-3-(methyldithio)furan	Meaty	1658	0	1	1.25
2-/3-Methylbutanoic acid	Cheese	1663	3	3	2.75
(E,E)-2,4-nonadienal	Fatty	1692	1.25	1.25	1
3-Methyl-2,4-nonandione	Floral	1711	0.5	0.5	0
Methionol	Boiled potato	1717	0	0.5	0
Dimethyltetrasulfide	Cabbage	1720	0	0	1.5
Unknown	Onion, gravy	1726	0.5	0.75	0.75
Pentanoic acid	Cheese	1731	0	0.75	0.5
(E)-2-undecenal	Metallic	1740	0	1	1
2-Acetyl-2-thiazoline	Roasty	1749	0	1	1
Ethyl-2-phenylacetate	Honey, floral	1757	0.5	0.5	0
Unknown	Mouldy, musty	1769	0	0	2
3-Methylpentanoic acid	Cheese	1771	0	0.5	1
2,4,6-Trichloroanisole	Mouldy	1794	1	0	0
2-Phenylethylacetate	Honey	1797	0	0.5	0
(E,E)-2,4-decadienal	Fatty	1803	0.75	1.25	1.5
(E)-β-damascenone, (E)-β-damascone	Apple	1809	0	0.5	1
Geosmin	Earthy	1812	0.5	0	1
2-Propionyl-2-thiazoline	Roasty	1821	0.5	0	0
Calamenene	Clove	1826	0	0.5	0
3-Mercapto-1-hexanol	Rhubarb	1832	1.5	1.25	1.25
Hexanoic acid	Goat	1835	0.5	1	0.5
Geraniol	Rose	1844	1	0.5	0.5
2-Hydroxy-3,4-dimethyl-2-cyclopenten-1-one	Caramel	1850	0	1	1
2-Methoxyphenol	Smoky	1853	1	1.5	2
(E,E,Z)-2,4,6-nonatrienal	Oatflakes	1868	1.5	0.5	1.25
2-Phenylethanol	Honey	1900	1	0	2
2-Ethyl-3-mercapto-1-hexanol	Meaty	1952	0	0	0.5
Trans-2,3-epoxydodecanal	Citrus, soapy	1958	0	0	1
Maltol	Caramel	1961	0.5	1.5	1.5
Trans-4,5-epoxy-(E)-2-decenal	Metallic	1997	2	1.5	1
4-Ethyl-2-methoxyphenol	Smoky	2016	0	0	0.5
p-anisaldehyde	Aniseed	2019	0	0	0.5
γ-Nonalactone	Peach	2023	1	1	0.5
Furaneol	Caramel	2026	2	2.25	1.75
Octanoic acid	Goat	2039	0	0	0.5
γ-(Z)-2-nonenolactone	Coconut	2065	0	0	0.5
3-/4-Methylphenol	Phenolic	2074	0.5	0.5	0.5
2,6-Dichlorophenol	Phenolic, medical	2100	0	0	0.75
γ-Decalactone	Peach	2133	1	1	1
Unknown	Perfume	2137	1	0.5	0.5
Eugenol	Clove	2157	1.25	1	1
3-/4-Ethylphenol	Phenolic	2173	0.5	0.5	0.5
2-Methoxy-4-vinylphenol	Clove	2187	0.75	1	1
Sotolon	Seasoning	2197	1.75	2.25	2.25
2-Aminoacetophenone	Foxy	2210	1	1.5	1.75
Decanoic acid	Soapy	2247	0	0	0.5
3-/4-Propylphenol	Phenolic	2257	0	0.5	0
Isoeugenol	Ham	2337	0	1	1
γ-Dodecalactone	Peach	2378	0	1.5	1.25
Coumarine	Woodruff	2448	0.5	0	0.5
3-Methylindole	Fecal	2485	0.5	0.5	0
Phenylacetic acid	Honey	2546	1.75	2.25	2
Vanillin	Vanilla	2562	1.5	2	1.5
Phenylpropanoic acid	Goat	>2600	0.5	1	0.5

^1^ Compounds were identified on the basis of their linear retention indices, odour qualities, and odour thresholds as perceived at the sniffing port. ^2^ Linear retention index. ^3^ Intensity: 0 = not detectable; 1 = weakly detectable; 2 = unequivocally detectable; 3 = intensely detectable; steps of 0.5 possible. ^4^ n.d. = not determined.
